# CrypticProteinDB: an integrated database of proteome and immunopeptidome derived non-canonical cancer proteins

**DOI:** 10.1093/narcan/zcad024

**Published:** 2023-06-01

**Authors:** Ghofran Othoum, Christopher A Maher

**Affiliations:** Department of Internal Medicine, Washington University School of Medicine, St. Louis, MO 63108, USA; Department of Internal Medicine, Washington University School of Medicine, St. Louis, MO 63108, USA; Department of Biomedical Engineering, Washington University in St. Louis, MO 63108, USA; Alvin J. Siteman Cancer Center, Washington University in St. Louis, St. Louis, MO 63108, USA

## Abstract

Translated non-canonical proteins derived from noncoding regions or alternative open reading frames (ORFs) can contribute to critical and diverse cellular processes. In the context of cancer, they also represent an under-appreciated source of targets for cancer immunotherapy through their tumor-enriched expression or by harboring somatic mutations that produce neoantigens. Here, we introduce the largest integration and proteogenomic analysis of novel peptides to assess the prevalence of non-canonical ORFs (ncORFs) in more than 900 patient proteomes and 26 immunopeptidome datasets across 14 cancer types. The integrative proteogenomic analysis of whole-cell proteomes and immunopeptidomes revealed peptide support for a nonredundant set of 9760 upstream, downstream, and out-of-frame ncORFs in protein coding genes and 12811 in noncoding RNAs. Notably, 6486 ncORFs were derived from differentially expressed genes and 340 were ubiquitously translated across eight or more cancers. The analysis also led to the discovery of thirty-four epitopes and eight neoantigens from non-canonical proteins in two cohorts as novel cancer immunotargets. Collectively, our analysis integrated both bottom-up proteogenomic and targeted peptide validation to illustrate the prevalence of translated non-canonical proteins in cancer and to provide a resource for the prioritization of novel proteins supported by proteomic, immunopeptidomic, genomic and transcriptomic data, available at https://www.maherlab.com/crypticproteindb.

## Graphical Abstract

**Graphical Abstract:**
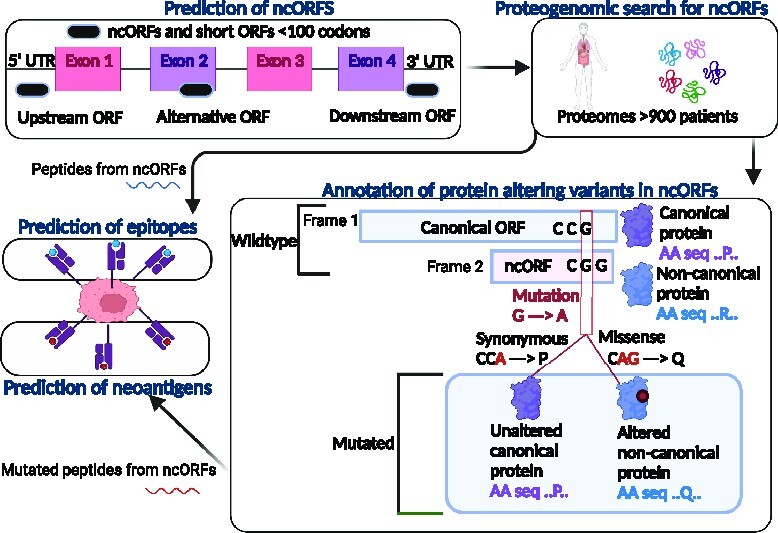

## INTRODUCTION

Recent studies have revealed functional small peptides encoded in long noncoding RNAs and circular RNAs with critical functions in cancer formation and progression (1–7). Two additional understudied classes of non-canonical open reading frames (ncORFs), with evidence of encoded peptides, include cryptic ORFs, defined as ORFs from (i) untranslated regions (UTRs) in annotated transcripts (8–10) and (ii) ORFs from alternative reading frames (11–13). Regulatory functions have been assigned to cryptic ORFs including reducing the translation efficiency of the main coding sequence (14), invoking mRNA decay (15–17) or alternatively, increasing translational efficiency under specific cellular conditions (18). This can be exemplified by well-characterized cryptic ORFs encoding biologically active proteins in cancer such as ORFs upstream of *CDKN2A* in melanoma (19), formed by a mutation giving rise to its initiation codon; *NPM1* in lymphomas and leukemias, showing increased ribosome occupancy (20); *ERCC5* in neuroblastoma, formed by a common polymorphic variant (9); and *CDKN1B* in a patient with pancreatic neoplasm, showing a mutation-driven phenotype (8).

Several recent studies have used ribosomal profiling or mass spectrometry to detect encoded peptides in non-canonical ORFs (ncORFs) (1,20–24). For instance, systematic analysis of ribosome profiling data showed that 40% of lncRNAs, 35% of upstream ORFs, and 4% of downstream ORFs are translated (21). Similar strategies have also been applied to discover major histocompatibility complex-bound peptides (MHC-peptides) from non-canonical sources (22–25) that contribute to an under-appreciated quantity of known targetable epitopes in cancer immunotherapy. Specifically, the discovery of major histocompatibility complex I (MHC I) peptides from retained introns in two melanoma cohorts increased the mean novel peptide load in addition to the load from canonical somatic peptides (24). Additionally, proteogenomic search of six-frame translation of the human genome in B lymphoblastoid cells revealed 10% of MHC peptides are from non-canonical regions in short ORFs translated into proteins with atypical C termini (23). More recently, analysis of Ribo-Seq data using 29 samples from B cells, glioblastoma, melanoma, colon carcinoma and melanocytes revealed 3555 translated novel ORFs presented on MHC I (22). Finally, integrating transcriptomics, ribosome profiling data, and proteomics from human B cell lymphomas showed that despite being shorter in length and less stable than canonical proteins, cryptic proteins are fivefold more efficient at generating MHC-I-associated peptides (26). Collectively, these studies highlight the value of using systematic and integrative analysis to evaluate the prevalence of translated ncORFs across cancer types and their enrichment with cancer relevant peptides. However, there is yet to be a study that integrates both global-proteomics and immunoproteomics available across a broader range of cancer tissues to detect non-canonical translation that could be missed with single cancer type studies, with proteogenomic databases that are limited by ribosome footprint evidence, or with studies that search proteomic samples only. In this study, whole-cell proteomes of 951 patients were analyzed across nine cancer types from the Clinical Proteomic Tumor Analysis Consortium (CPTAC) resource (27), in addition to other publicly available immunopeptidome datasets, to provide the largest pan-cancer atlas of ncORFs and novel MHC-peptides. We specifically focused on cryptic ORFs from 5′ and 3′ UTRs and alternative frames as well as ORFs from long noncoding RNAs, pseudogenes, miscellaneous RNAs and ribosomal RNAs, including short ORFs that are less than 100 codons (Supplementary Figure S1). First, using 642262 predicted ncORFs, proteome and immunopeptidome samples showed that 2.2% of the predicted ncORFs were supported by at least one peptide. We further focused on 340 ncORFs that showed evidence of translation in eight or more cancers, indicating possible pervasive translation across cancer types and suggesting that these ncORFs might be essential for cancer associated phenotypes. Additionally, 6486 ncORFs were in genes that were differentially expressed in cancer suggesting potential relevance in tumorigenesis. Thirty-four peptides in cryptic ORFs from proteome samples were identified with high binding affinity to MHC (hereinafter referred to as epitopes) that hold promise as potential immunotherapy targets. Additionally, eight MHC-bound peptides were identified in ncORFs derived from somatic mutations (hereinafter referred to as neoantigens), with supporting mutated peptides that overlapped canonical ORFs but would have eluded existing neoantigen discovery pipelines since they produced silent mutations. Additionally, proteogenomic analysis of 18 991 018 MS/MS from 26 immunopeptidome datasets yielded 9258 MHC-I peptides (hereinafter referred to as immunopeptides). The prevalence of epitopes and immunopeptides from translated ncORFs across patient proteomes and beyond mutated protein sequences from canonical coding sequence regions shows that these non-canonical targets are highly effective in eliciting an immune response in multiple patients, a property often desired in immunotargets. Altogether, the integrative analysis provided in CrypticProteinDB of novel protein coding sequences reveals their prevalent translation in cancer, with potential functional roles. It also highlights the potential strength of ncORFs with MHC bound peptides, stressing the importance of exploring more diversified sources of cancer epitopes beyond neoantigens from canonical proteins, especially in patients with low tumor mutational burden. The integrative analysis and generated data are provided at CrypticProteinDB (https://www.maherlab.com/crypticproteindb).

## MATERIALS AND METHODS

### Global proteomic analysis of ncORFs

To construct the database for the proteogenomic search, predicted 3′ UTR ORFs, 5′ UTR ORFs and alternative ORFs as well as ORFs from noncoding RNAs, miscellaneous RNAs and ribosomal RNAs were used from OpenProt v 1.6 (28), utilizing translation-related features such as the presence of Kozak motifs, high efficiency translation initiation sites, and predicted protein domains.

For the proteomic samples, we utilized 8690 mzML files from the CPTAC portal https://cptac-data-portal.georgetown.edu/ representing eight cancers: oral squamous cell carcinoma (OSCC), hepatocellular carcinoma (HCC), lung adenocarcinoma (LUAD), clear cell renal carcinoma (CCRC), breast cancer (BRCA), uterine corpus endometrial carcinoma (UCEC), colon cancer (CRC) and ovarian cancer (OV) and 107 mzML from prostate adenocarcinoma patients (PRAD) (29) from ftp://massive.ucsd.edu/MSV000081552.

For the proteogenomic search we used a global false discovery rate (FDR) control approach followed by peptide centric validation as this showed the highest sensitivity in similar data (30). First, the proteogenomic database was appended with 20549 canonical sequences from Uniprot release 2021-02 (31) to account for canonical peptides as well as reversed target sequences without adding any further contaminants. The target-decoy approach was used to derive peptide spectrum matches (PSMs) with a FDR threshold of <0.01. Given the large size of the proteomic search space, the parallel feature of the *MSFragger* search engine (32) was used, followed by PSM postprocessing using the *Philosopher* pipeline (https://github.com/Nesvilab/philosopher) v.3.4.13 (33). Specifically, *PeptideProphet* was used with semi-parametric mixture modeling to assign probabilities to the identified PSMs with subsequent filtering using a 1% FDR threshold at the PSM level. PepQuery v1.3.0 was used to validate the peptides with a threshold of ≤0.01 for *P* value and excluding all peptides with hits using the unrestricted post-translational modification search. Further, BLASTP (BLAST + 2.13.0) was used to eliminate all proteins with alignment to canonical proteins from GENCODE (v. 39) with an E value less than 0.01. *frequent* and *labelquant* from the *Philosopher* pipeline v.3.4.13 were used to quantify the intensities of proteomic samples in cohorts for both iTRAQ and TMT labelling. Cohorts where label-free quantification was used were not included to avoid skewed statistical analysis due to a large discrepancy in the number of PSMs generated compared to iTRAQ and TMT.

For the proteogenomic search, semi-tryptic peptides, two missed cleavage sites, and C12/C13 isotope errors were allowed, and the precursor-ion mass tolerance was set to 20 ppm. Recommended post-translational modifications from CPTAC were used for each labelling method as indicated in Table 1.

**Table 1.** Allowed post-translational modifications in the experimental proteogenomic design of TMT and iTRAQ experiments

Protocol	Modification(s)	Modification type
TMT	Cystine carbamidomethylation (+57.0215) and Lysine TMT labeling (+229.1629)	Fixed
TMT	Methionine oxidation (+15.9949), N-terminal protein acetylation (+42.0106), and TMT labeling of peptide N terminus and serine residues	Variable
iTRAQ	Cysteine carbamidomethylation (+57.0215), iTRAQ labeling of lysine (+144.10253) and peptide N terminus	Fixed
iTRAQ	Methionine oxidation (+15.9949)	Variable

For immunopeptidome analysis, the following studies were included from PRIDE (Proteomics Identification Database): PXD000394 for colorectal cancer; PXD009754, PXD009755, PXD009752 and PXD009935 for lung cancer; PXD008984, PXD003790, and PXD008127 for glioblastoma; PXD009738 and PXD000394 for breast cancer; PXD008937, PXD011766 and PXD004894 for melanoma; PXD005704, PXD010808 and PXD004746 for lymphoma; PXD009751, PXD009749, PXD009750, PXD009753, PXD010808, PXD000394, PXD012083 and PXD007935 for leukemia; and PXD009925 and PXD006939 for meningioma. To perform the search, first MSconvert from ProteoWizard v.3.0.19014 was used to convert the MS/MS from RAW to MGF formats. A custom database that included the non-canonical sequences, UniProt sequences (release 2021-02) and decoy reverse sequences was used for the search with the following parameters: carbamidomethyl as a fixed modification and oxidation [M] as a variable modification, no enzyme specificity, a precursor mass tolerance of 10 ppm and peptide length range of 8–25 aa. Only peptides with computed probability ≥0.99 were retained and considered confident matches.

For conservation analysis, we used the inferred conservation relationships from an all-vs-all BLAST search in OpenProt between ten species, including humans, for which OpenProt has ORF predictions: *Pan troglodytes*, *Mus musculus*, *Rattus norvegicus*, *Bos taurus*, *Ovis aries*, *Danio rerio*, *Drosophila melanogaster*, *Caenorhabditis elegans* and *Saccharomyces cerevisiae*.

Hydrophobicity index was predicted using SSRCalc vQ.0 (34) with 100 Å C18 column option, 0.1% formic acid separation system and without cysteine protection.

The absolute difference between predicted and observed retention time was used to evaluate the retention time error. For the predicted value, autoRT (30) was used. The transfer learning module was utilized to train the model on The Cancer Genome Atlas (TCGA) OV and TCGA BRCA CPTAC processed data of canonical peptides. Wilcoxon rank sum test was used to identify significantly variant characteristics of the identified peptides including length, peptide probability and conservation across different ncORFs and in comparisons between peptide encoding and non-encoding proteins.

### Protein coding genes differential expression analysis

Pre-aligned RNA-Seq bam files were utilized from TCGA for tumor and adjacent normal pairs for the following cancers: breast invasive carcinoma, head and neck squamous cell carcinoma, kidney renal clear cell carcinoma, liver hepatocellular carcinoma, lung adenocarcinoma, prostate adenocarcinoma, and uterine corpus endometrial carcinoma. To evaluate if genes associated with PSM-supported ncORFs were differentially expressed in tumor and normal pairs, the negative binomial model from edgeR version 3.11 (35) was used. Genes with significant *P* value (<0.01) and log_2_-fold change ≥1.5 were considered over-expressed and genes with log_2_-fold change ≤−1.5 were considered under-expressed in the tumor sample.

### Epitopes and neoantigens discovery from proteome samples

We downloaded HLA genotyping for samples from the TCGA breast and ovarian cancer cohorts, predicted through Optitype (v1.3.1) (36), from the GDC portal (https://gdc.cancer.gov/about-data/publications/panimmune) (37). NetMHCpan v 4.1 (38) was used to predict the peptide-HLA allele binding affinity using a high binding affinity threshold (≤150 nM).

To analyze somatic mutations, files in mutation annotation format (MAF) for TCGA BRCA, TCGA OV, CPTAC-2 and CPTAC-3 were downloaded from Genomic Data Commons (GDC) portal (https://portal.gdc.cancer.gov/). The consequence of each variant was determined using the Ensembl Variant Effect Predictor (VEP, version 98.2) (39) based on the hg38 assembly and using the custom annotation feature.

To identify neoantigens, whole exome sequencing data and proteomic datasets of the TCGA breast and ovarian cancer cohorts were used to generate novel protein sequences for each patient using all silent mutations of predicted ncORFs. Silent mutations in canonical coding regions affecting out-of-frame ncORFs as well as mutations not overlapping canonical coding regions primarily affecting upstream or downstream ncORFs were included to generate the mutated proteome. To avoid false positives caused by hits emerging from wildtype peptides, we first eliminated all spectra with hits to wildtype peptides from the search space.

## RESULTS

### A comprehensive and integrated proteogenomic search for peptides from ncORFs

**Figure 1.**
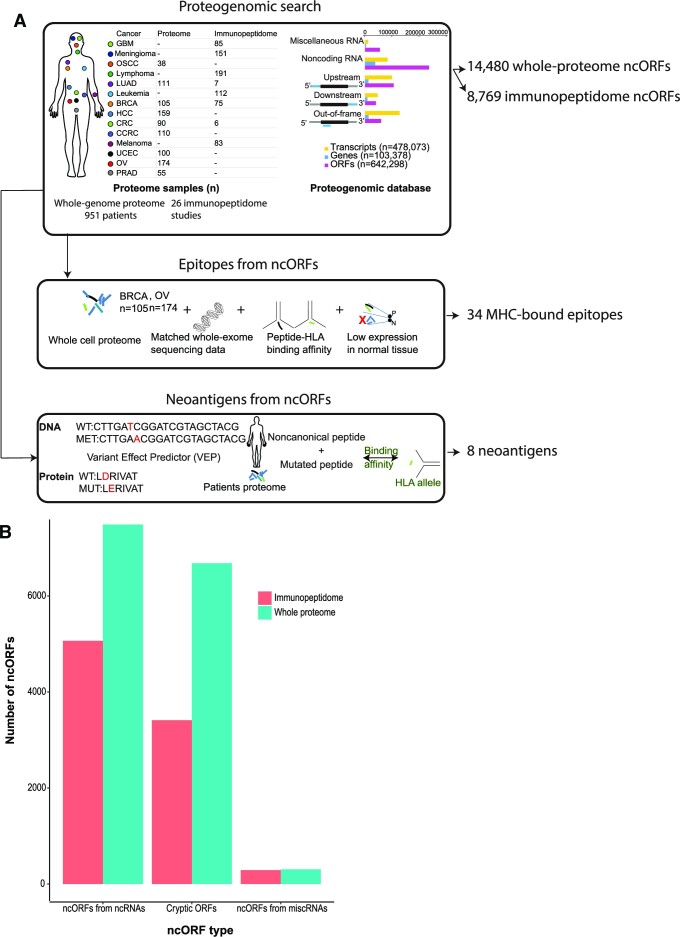
Integrated proteogenomic analysis of whole-cell proteomes and immunopeptidomes allows the identification of cryptic ncORFs translated across cancers. (**A**) A flowchart showing the workflow used for the analysis: whole-genome proteomes of 951 patients and 26 immunopeptidome samples for the following cancer cohorts: oral squamous cell carcinoma (OSCC), colorectal cancer (CRC), hepatocellular carcinoma (HCC), lung adenocarcinoma (LUAD), clear cell renal carcinoma (CCRC), prostate adenocarcinoma (PRAD), breast cancer (BRCA), uterine corpus endometrial carcinoma (UCEC), glioblastoma multiforme (GBM), melanoma, lymphoma, leukemia, meningioma, and ovarian cancer (OV). For the proteogenomic search database we included upstream, downstream and out-of-frame ORFs from transcripts of protein coding genes as well as ORFs from noncoding RNAs including: lncRNAs, pseudogenes and miscellaneous RNAs. The proteogenomic search was followed by peptide-centric validation, yielding 8769 ncORFs. To identify MHC-bound epitopes, peptides from whole-genome proteomes from BRCA and OV were prioritized based on their HLA-binding affinity and low expression in normal tissue, yielding 34 epitopes. To prioritize neoantigens, mutated novel proteins where search against whole-genome proteomes from BRCA and OV, followed by peptide-centric validation, yielding eight MHC bound neoantigens. (**B**) Number of peptide supported ncORFs from whole-genome proteomes and immunopeptidomes.

Conventionally, tandem mass spectrometry (MS/MS) data searches use canonical sequences of annotated proteins thereby overlooking putative peptides derived from ncORFs. To address this, we searched for peptides from ncORFs using whole-cell proteomic samples from nine diverse cancers: oral squamous cell carcinoma, hepatocellular carcinoma, lung adenocarcinoma, clear cell renal carcinoma, prostate adenocarcinoma, breast cancer, ovarian cancer, colon cancer, and uterine corpus endometrial carcinoma. To identify immunopeptides, we analyzed 26 MS-based immunopeptidomes spanning eight cancers: breast cancer, lung cancer, colon cancer, lymphoma, leukemia, meningioma, glioblastoma, and melanoma; and including cell lines: HCT-116, HCC1143, HCC1937, A375 and A549 (Figure 1A). For the proteogenomic search, a two-tier approach was used, starting with a target-decoy search utilizing a custom database of ncORFs appended to canonical proteins and reversed sequences as decoys; followed by targeted peptide validation where other peptide-centric statistical scores were used to filter out false positives. We opted to use this combination of peptide validation (i.e. global FDR followed by peptide centric validation) as recent analysis in similar cohorts showed that this approach is sufficiently sensitive with minimal compromise to the accuracy at which novel peptides are identified, especially compared to other validation methods such as two-stage or separate FDR (30) (Supplementary Figure S2). For the search, we used 642 262 ncORFs (between start and stop codons) from OpenProt v1.6; 267 006 from untranslated and out-of-frame regions in protein coding genes (defined as cryptic ORFs), 303 777 from noncoding RNAs (lncRNAs, pseudogenes and novel isoforms) and 71 479 ORFs from miscellaneous RNAs. Of the 267 006 cryptic ORFs in transcripts of protein coding genes, 31.1% are out-of-frame (4 535 104 amino acids aa), 47.8% reside in 3′UTR ORFs (6 969 354 aa), and 21.1% reside in 5′ UTR ORFs (2 884 072 aa).

Proteomic analysis revealed 14480 ncORFs had support from at least one peptide across nine cancers and 8769 ncORFs had support from 26 immunopeptidome datasets. In total, 22576 nonredundant ncORFs had peptide support from proteomes and immunopeptidomes (available for download at CrypticProteinDB; Figure 1B). We compared the lengths of ncORFs with peptides to ncORFs without peptides and found that peptide supported ncORFs are longer than those without (median is 52 for peptide-supported ncORFs and 47 for without support) (*P* value <2.2e−16, Wilcoxon rank sum test) (Supplementary Figure S3). We subsequently used peptide-supported ncORFs to determine: (i) if cryptic ncORFs are pervasively translated and may play a role in cancer or (ii) if cryptic ORFs with MHC bound peptides from whole proteomic analysis produce cancer MHC-bound peptides or are derived from somatic mutations.

### Integrative proteogenomic analysis of ncORFs reveals ubiquitously translated novel proteins from differentially expressed cancer genes

To identify proteins from ncORFs that are relevant to cancer functions, we focused on non-canonical proteins from genes directly or indirectly implicated in cancer as catalogued the Cancer Gene Census (CGC) (40), supported by multiple peptides, with an ortholog in at least one more species, and a high sequence-based predicted coding potential from CNIT (41). We additionally used RNA-Seq data from The Cancer Genome Atlas (TCGA) (42) to confirm that just like canonical proteins (43), genes harboring peptide-supported ncORFs showed a high ratio of differentially expressed genes (44.8%). NcORFs from differentially expressed cancer genes include an out-of-frame ncORFs from *NKX2-1* with a CNIT score of 0.8. However, it was not differentially expressed in lung cancer (the cohort that had the peptide). Another case is an ncORF in *ZBTB16* (CNIT score 0.7), which was differentially expressed in three of the cancers which samples had peptide support (BRCA, EOGC, CCRC).

**Figure 2.**
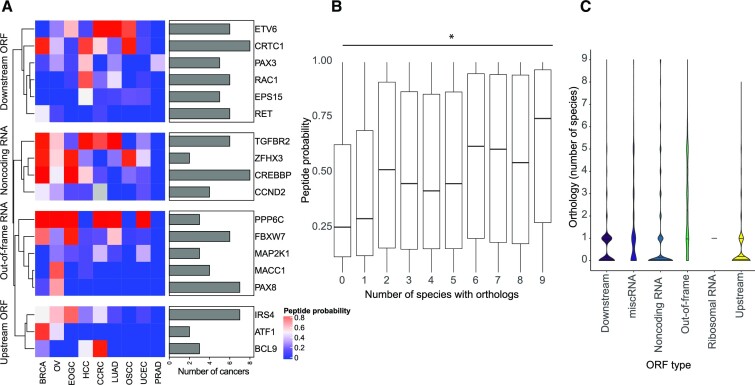
Conserved ncORFs are abundant in transcripts of cancer genes. (**A**) Many cancer hallmark genes are identified with at least two peptides across cancers for each type of novel protein, highlighting the use of proteogenomic analysis to identify novel cancer proteins. (**B**) Analysis of variations in the number of orthologs of ncORFs across species shows that novel proteins which are conserved across more species have peptides identified with higher posterior probability compared to proteins conserved in less species (Wilcoxon rank sum test *P* value < 2e−16). (**C**) LncRNAs and out-of-frame ORFs encode more proteins with orthologs in other species compared to other types of noncanonical proteins (Wilcoxon rank sum test *P* value < 22–16).

While these proteomic data sets may under-represent ncORFs since they do not reach saturation, we assessed the prevalence of identified cryptic ORFs across the patient proteomes. Notably, 340 ncORFs were pervasively translated in eight or more cancers. Exemplary cases include ncORFs in the cancer hallmark genes *CREBBP, CRTC1, CSF3R, FGFR2, IKZF1* and *MAP2K2* with peptide support in all but two of the analyzed cancer types (Figure 2A).

We further investigated the conservation level of noncanonical proteins, represented in the number of species with an ortholog of the protein, as a metric for evaluating the quality of the peptide-spectrum matches. Analysis showed that proteins with orthologs in six or more species had PSMs that had significantly higher posterior probability compared to those with no orthologs or with orthologs in five or fewer species (*P* value <2.22e−16, Wilcoxon rank sum test) (Figure 2B). Since it is expected that conserved proteins are longer in length, we compared how the conservation level of the proteins correlated with protein length. The analysis showed that proteins conserved in six or more species are longer in length than proteins that are conserved in five or less species (mean: 92.3 and 63.3 aa respectively, *P* value 6.58e−25, Wilcoxon rank sum test) (Supplementary Figure S4). We further compared the conservation level across the noncanonical protein categories showing that cryptic ORFs in upstream and downstream untranslated regions have slightly better conservation levels compared to other types (Figure 2C). Out-of-frame proteins with partial or complete overlap with canonical proteins expectedly had higher conservation levels compared to all other categories (*P* value < 2.22e−16, Wilcoxon rank sum test). However, despite its importance in providing support to high confidence peptides, conservation alone is not independently sufficient to indicate translations as thousands of ncORFs have been found without conservation evidence (44,45).

### Evaluation of ncORFs as a source of epitopes from whole proteome samples

**Figure 3.**
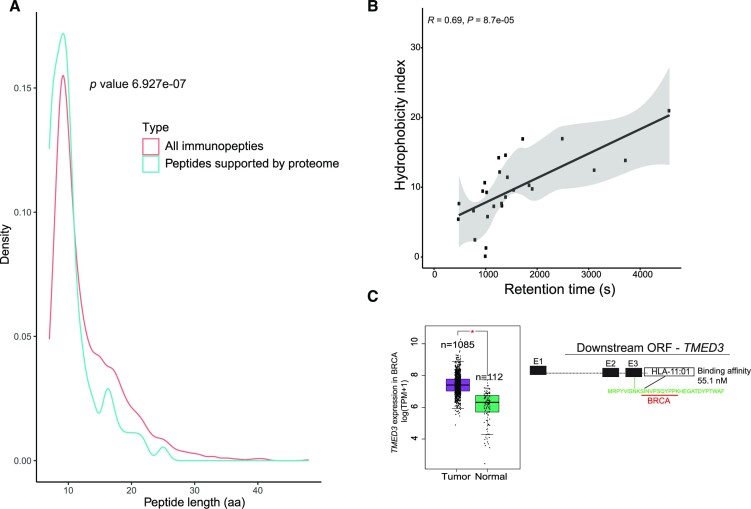
Features of peptides from whole-cell proteomes and immunopeptidomes in ncORFs. (**A**) Peptides validated in both immunopeptidomes, and global proteome samples are shorter in length compared to peptides identified in immunopeptidome samples only. Shown *P* values from Wilcoxon rank sum test. (**B**) Correlation between predicted hydrophobicity index and observed mean retention time for epitopes from whole-cell proteomes that show low retention time error (<5 min) and high binding affinity (<150 nm). (**C**) An exemplary case of an epitope identified in a breast cancer proteome sample in the 3′ UTR of *TMED3*, a differentially expressed gene in breast cancer.

The large integrative nature of proteomes (identifies all peptides) and immunopeptidomes (immunopeptides) used in this study created an opportunity to assess how many of the peptides identified from both sources are in fact shared. Specifically, we used 8769 non-canonical proteins with at least one high probability peptide from an immunopeptidome sample and searched for peptide support in whole proteome samples. On average, across ncORF types, 2.8% of immunopeptidome peptides and 7.6% of immunopeptidome-supported ncORFs were supported by a peptide in a proteome sample. It was noted that peptides from ncORFs identified in both proteomes and immunopeptidome samples had significantly shorter peptides (*P* value < 2.2e−16, Wilcoxon rank sum test) (Figure 3A). The shorter length of peptides supported in both immunopeptidomes and proteomes is expected as class I immunopeptides are usually short ranging from 8 to 11 amino acids in length (46).

Since immunopeptidome data was not always available, we focused on identifying epitopes from proteome samples. Specifically, we evaluated the discovery rate of MHC-bound peptides from whole-cell proteomic samples, focusing on peptides showing high binding affinity to MHC in samples from TCGA cohorts with transcriptomic, proteomic, and HLA-genotyping datasets available (BRCA and OV cohorts). MHC binding prediction on 6682 cryptic peptides from ncORFs in protein coding genes (5′UTR, 3′ UTR and out-of-frame ORFs) showed 34 proteome-derived peptides with high binding affinity to MHC molecules (available for download from CrypticProteinDB).

To evaluate the quality of the identified non-canonical MHC-bound peptides, we considered variation between predicted sequence-based retention time (RT) and observed retention time (24), resulting in a total of 13 epitopes (38%) with retention time error less than five minutes (Figure 3B). Additionally, since the proteogenomic search was not restricted to ncORFs from tumor specific transcripts, we also considered nine epitopes (MHC-bound peptides) from ncORFs in lowly expressed genes in normal tissue from GTEx (median expression across samples: <1 transcript per million) in the same tissue in which the peptide is identified as a more accurate subset of MHC-bound peptides, yielding a total of 18 MHC-bound peptides supported by at least one of these filters (i.e. low retention time error and low expression in GTEx tissues).

An example of a non-canonical MHC-bound peptide from a cancer-relevant gene is an 11 aa-long MHC bound peptide (binding affinity 8.91 nM) from a downstream ncORF in *TMED3*, an upregulated gene in breast cancer promoting proliferation, migration, and invasion of breast cancer cells (47) (Figure 3C). Another example is an epitope in an out-of-frame ORF in *CALCR*—a Calcitonin receptor gene—in ovarian cancer (KVFGLNILK), that had a high MHC-I binding affinity (8.91 nM) and was conserved in five species (Supplementary Figure S5).

We further compared non-canonical MHC-bound peptides from the ovarian and breast cancer cohorts to previously identified neoantigens from canonical sequences (37) and to those predicted in the TSNAdb database of predicted neoantigens in TCGA cohorts (48). For the comparison, we considered all predicted canonical neoantigens with and without MS peptide support. First, prior to peptide validation and considering all peptide-HLA hits with the standard NetMHCpan rank of less than two, there were 73 ncORFs with MHC-bound peptides from genes that have no known or predicted neoantigens from canonical proteins in the studied cancers (Supplementary Figure S6). Of this list, 17 ncORFs had MHC-bound peptides with MHC binding affinity <150 nm and were validated by PepQuery. This analysis confirms that the prediction of non-canonical MHC-bound peptides increases the repertoire of targetable epitopes in genes that have been overlooked for their functional relevance in cancer.

### Proteogenomic analysis of mutated non-canonical peptides from whole proteome samples for the discovery of neoantigens

**Figure 4.**
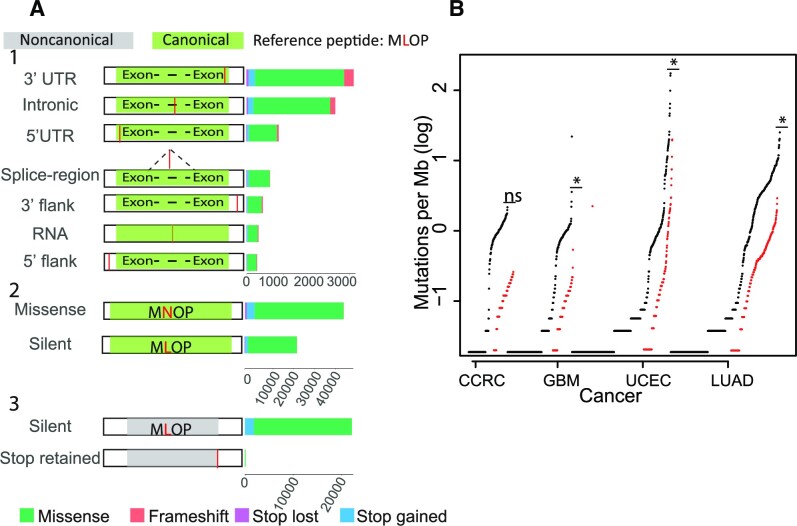
Mutations in ncORFs have a mutational burden comparable to that of canonical proteins. (**A**) Summary of variants causing novel translated protein sequence of predicted ncORFs in the following classifications: 1. Mutations from untranslated regions causing alterations in protein sequences of ncORFs. The original variant annotations in canonical sequences are: 3′–5′ UTR, 3′–5′ flank, RNA, intron, and splice region. The consequential non-canonical variant annotations are frameshift deletion/insertion, in-frame deletion/insertion, missense, or a gained/lost stop. 2. Mutations from overlapping canonical and non-canonical sequences annotated as missense or silent in canonical sequences. 3. Missense mutations which do not alter translated protein in canonical sequences and overlapping ncORFs. (**B**) Tumor mutation burden plot for all canonical mutations (black) and for the subset of mutations that are only consequential in non-canonical sequences (red) in CPTAC cohorts Asterisks represent a Wilcoxon rank sum test *P* value < 2.22e−16.

Here, we wanted to investigate if point mutations that alter non-canonical protein sequences could generate neoantigens that elicit immune responses in patients with the mutations. First, a total of 14 211 somatic variants in CPTAC cohorts annotated as 3′–5′UTR, 3′–5′ flank, intron, splice region and RNA mutations were filtered to 8828 variants that caused an alteration in the translated protein sequence of a ncORF: 7560 caused a missense, 559 caused a frameshift, 549 caused a gained stop and 160 led to losing a stop variant (Figure 4A1).

Focusing on cryptic ncORFs in protein coding genes, there were 72 226 non-canonical, protein-altering variants in regions common to both canonical and non-canonical sequences—such as out-of-frame ncORFs or ORFs from two isoforms—47 174 from missense mutations in canonical sequences and 25 052 from silent mutations (Figure 4A2). Conversely, there were 28457 canonical protein-altering variants that did not alter the overlapping ncORF (28 229 were silent and 228 were retained stop variants) (Figure 4A3).

These results motivated investigating the variation between the mutational burden for each cohort using all somatic mutations in canonical proteins (causing missense, stop gained/lost, frameshift deletion/insertion and in-frame deletions/insertions) to those causing similar protein alterations in ncORFs. Although most of the cohorts had similar mutational frequencies for both canonical and ncORF sequences, the distribution of non-canonical variants per megabase (v/mb) compared to the canonical distribution varied significantly in some cancers (*P* value 8.5e−05 for GBM and 0.0018 for LUAD, Wilcoxon rank sum test) (Figure 4B).

To generate the mutated proteome for the proteogenomic analysis, whole exome sequencing data was integrated with the proteomic datasets to reannotate the mutations using all predicted ncORFs and perform the proteogenomic search. The analysis primarily included silent mutations in canonical coding regions affecting out-of-frame ncORFs, as well as mutations not overlapping canonical coding regions affecting upstream or downstream ncORFs. To avoid false positives caused by hits emerging from wildtype peptides, all spectra with hits to wildtype peptides from the search space were eliminated. Sample-matched mutated peptides and nonspecific matches were then used to predict HLA-peptide binding affinity for neoantigen identification. The analysis yielded a total of eight mutated peptides in non-canonical proteins (five from both breast and ovarian cancers), 25% of which were supported by other peptides.

## DISCUSSION

Here, we generated the largest atlas of novel proteins in cancer proteomes by integrating whole-cell proteomes, immunopeptidomes, HLA-genotyping, and transcriptomics data. A critical challenge with novel protein identification is that often relying on any single method for evidence of translation may not be sufficient to infer that a protein is synthesized. For instance, a dense ribosomal footprint is not direct evidence of translation and proteomic support of one peptide from a protein does not always result in a protein translated and synthesized (49). Additional challenges are created when using a large database for the proteogenomic search as it subjects the search to many false positives; resulting in inaccurate false discovery estimations. Here, we circumvented these challenges by using peptide-centric validation along with other orthogonal evidence from transcriptomics data, conservation of sequences across species, coding potential of predicted ORFs and/or MHC presentability. This allowed for a sensitive approach for discovering putative peptides without using stringent search filters (e.g. high peptide probability) or using an evidence-restricted database such as those supported by Ribo-Seq or RNA-Seq. This is particularly useful since a database derived solely from matched Ribo-Seq would not allow for the integration of proteomic samples across different tissues. Further, the close genomic proximity of short ncORFs in UTR regions and alternative frames to annotated proteins limits the effectiveness of a database derived from RNA-Seq.

This study represents the first systematic analysis using cancer proteomic datasets (CPTAC) to generate a comprehensive source of mapped peptides in varied classifications of ncORFs. Specifically, our analysis led to the identification of 6682 and 3412 cryptic proteins from whole-cell proteomes and immunopeptidomes, respectively; 7488 and 5066 non-coding RNAs from whole-cell proteomes and immunopeptidomes, respectively; and 307 and 289 miscRNAs from whole-cell proteomes and immunopeptidomes, respectively.

Focusing on ncORFs with putative functions in cancer, we identified 406 ncORFs in differentially expressed genes that were supported by peptides across different cancers, highlighting the potential that these proteins have key functions. Although we selected a minimum of eight cancers to establish pervasive translation, the actual number of samples with peptide support could be more, as sampling saturation cannot be reached in proteomic analysis.

By integrating immunopeptidome data with all available samples from CPTAC, the number of overlapping ncORFs between proteome and immunopeptidome samples was 7.6% at the protein level. Driven by the low number and limited tissue diversity of matched immunopeptidomes and whole-exome sequencing for primary/normal samples, we successfully showed how increasing the number of searchable whole-cell proteome samples enabled the discovery of 34 proteome-derived MHC-bound peptides and eight somatic mutation MHC-bound peptides. Compared to whole-cell proteome derived neoantigens from canonical sequences, the numbers of MS/MS neoantigens in ncORFs was not significantly different (e.g. there were 31 mutation MHC-bound peptides in canonical sequences from the breast cancer cohort using the Comet search engine and a two-stage FDR strategy (30)).

Our approach also highlights a very critical and often overlooked value of silent and UTR mutations for generating novel neoantigens. The majority of previously implemented neoantigen prediction pipelines have two significant limitations. First, these strategies filter silent mutations that may alter cryptic ORFs. Second, they focus on somatic variants residing within canonical proteins thereby excluding cryptic ORFs. However, we identified a significant number of silent mutations (33 880: 25 052 from regions overlapping canonical proteins and 8828 from UTR regions) causing an alteration in the translated proteins of non-canonical ORFs. These variants indicate that silent mutations should be used to discover novel MHC-bound peptides in ncORFs from different cancers. Overall, the three types of cryptic ORFs had a similar average number of mutations (1.4 for 3′ UTR proteins and 1.5 for 5′ UTR and out-of-frame proteins), consistent with the mean mutation frequency reported previously for these regions in cancer (50). Additionally, the number of non-canonical MHC-bound peptides from translated ncORFs was more than that from mutated peptides confirming previous reports (23) that non-canonical proteins are more enriched with ncORF derived MHC-bound peptide than mutation-derived ones. Expectedly, MHC-bound peptides from non-mutated proteins are more shared between samples than neoantigens (i.e. from mutated proteins), creating targetable MHC-bound peptides across a larger number of patients. One striking example is that of an MHC-bound peptide in an upstream ORF of the *EIPR1* gene, supported by peptides from the proteogenomic search in nine ovarian cancer samples. This shows how MHC-bound peptides from ncORFs could create better therapeutic targets that could elicit immune response in multiple patients. By identifying these novel tumor MHC-bound peptides from sequences usually not included in MHC-bound peptide discovery pipelines, either from cancer-specific ncORFs or from somatic mutations, we contributed to increasing the current knowledge of the true richness of the cancer immune repertoire. Future work should validate the MHC presentation, immunogenicity, and clinical importance of the MS derived MHC-bound peptides.

Collectively, we evaluated the true prevalence of novel proteins in cancer proteomes showing their enrichment in aberrantly expressed protein-coding genes, including those in genes lacking any known targetable neoantigens derived from canonical sequences and thus previously overlooked for their relevance in cancer and the development of immunotherapy targets.

## DATA AVAILABILITY

All data is available at https://www.maherlab.com/crypticproteindb.

## Supplementary Material

zcad024_Supplemental_File
