# Supplementary material for: CrypticProteinDB: an integrated database of proteome and immunopeptidome derived non-canonical cancer proteins
Source: NAR Cancer. 2023 Jun 1;5(2):zcad024. doi: 10.1093/narcan/zcad024 (PMC10233886; doi:10.1093/narcan/zcad024)

**Supplementary Fig S1**.A cartoon depiction of the positions of ncORFs, including short ORFs that are less than 100 codons in length. ncORFs and sORFs are either upstream of the canonical protein, downstream of the canonical protein, in an alternative open reading frame to that of the canonical protein or in a noncoding RNA. Black bars are exons and dashed lines are introns.

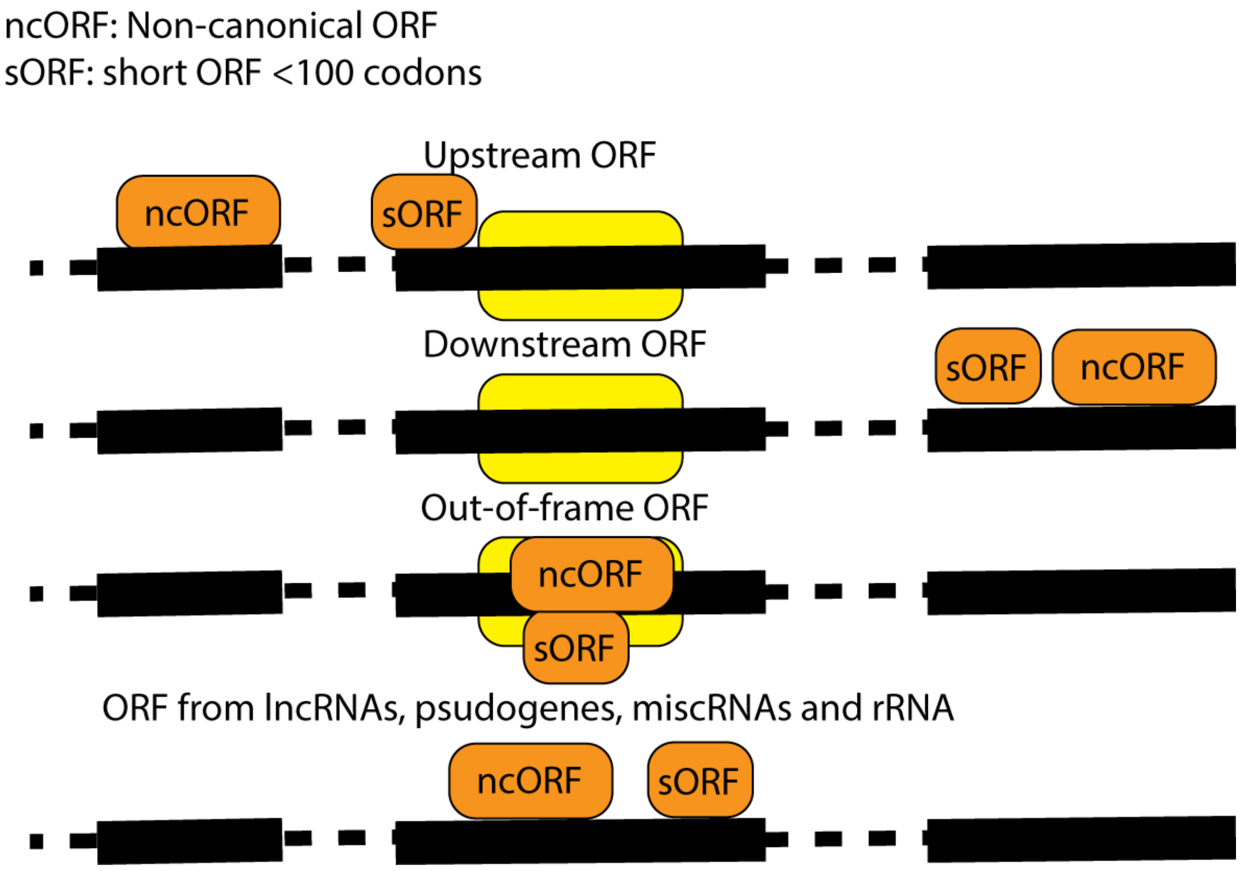

**Supplementary Fig S2.** A flowchart showing the workflow used for the analysis starting by proteogenomic identification and peptide-centric validation of peptides from 951 whole-genome proteomes and 26 immunopeptidome. For the proteogenomic search database we included upstream, downstream and out-of-frame ORFs from transcripts of protein coding genes as well as ORFs from noncoding RNAs including: lncRNAs, pseudogenes, and miscellaneous RNAs. To identify MHC-bound epitopes, peptides from whole-genome proteomes from BRCA and OV were prioritized based on their HLA-binding affinity, yielding 34 epitopes. To prioritize neoantigens, mutated novel proteins where search against whole-genome proteomes from BRCA and OV, yielding eight MHC bound neoantigens.

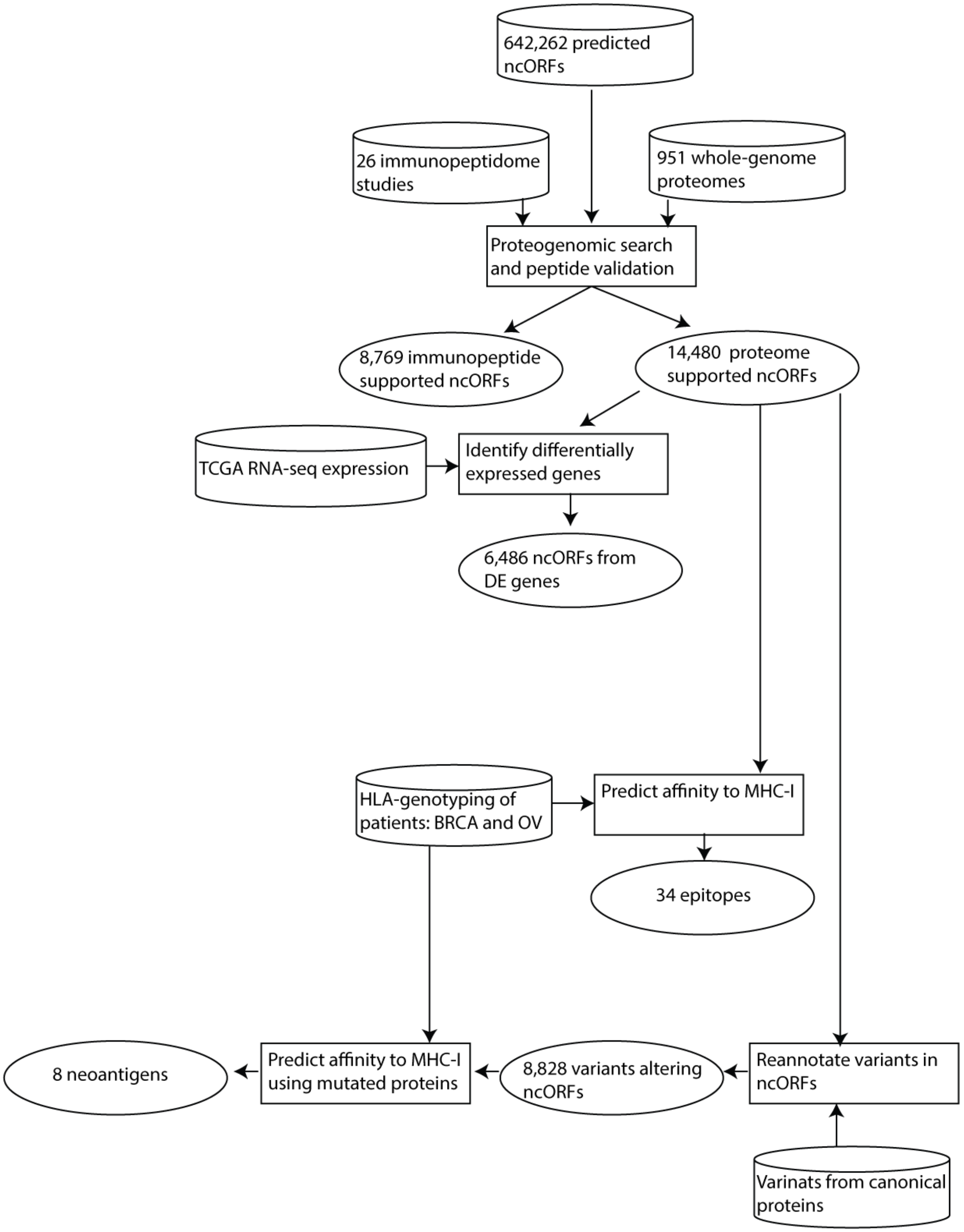

642,262 predicted ncORFs
26 immunopeptidome studies
951 whole-genome proteomes
Proteogenomic search and peptide validation
8,769 immunopeptide supported ncORFs
14,480 proteome supported ncORFs
TCGA RNA-seq expression
Identify differentially expressed genes
6,486 ncORFs from DE genes
HLA-genotyping of patients: BRCA and OV
Predict affinity to MHC-I
34 epitopes
8 neoantigens
Predict affinity to MHC-I using mutated proteins
8,828 variants altering ncORFs
Reannotate variants in ncORFs
Varinats from canonical proteins

**Supplementary Fig. S3**. Peptide supported ncORFs are longer than those without (median is 52 for peptide-supported ncORFs and 47 for without support) (P value < 2.2e-16, Wilcoxon rank sum test).

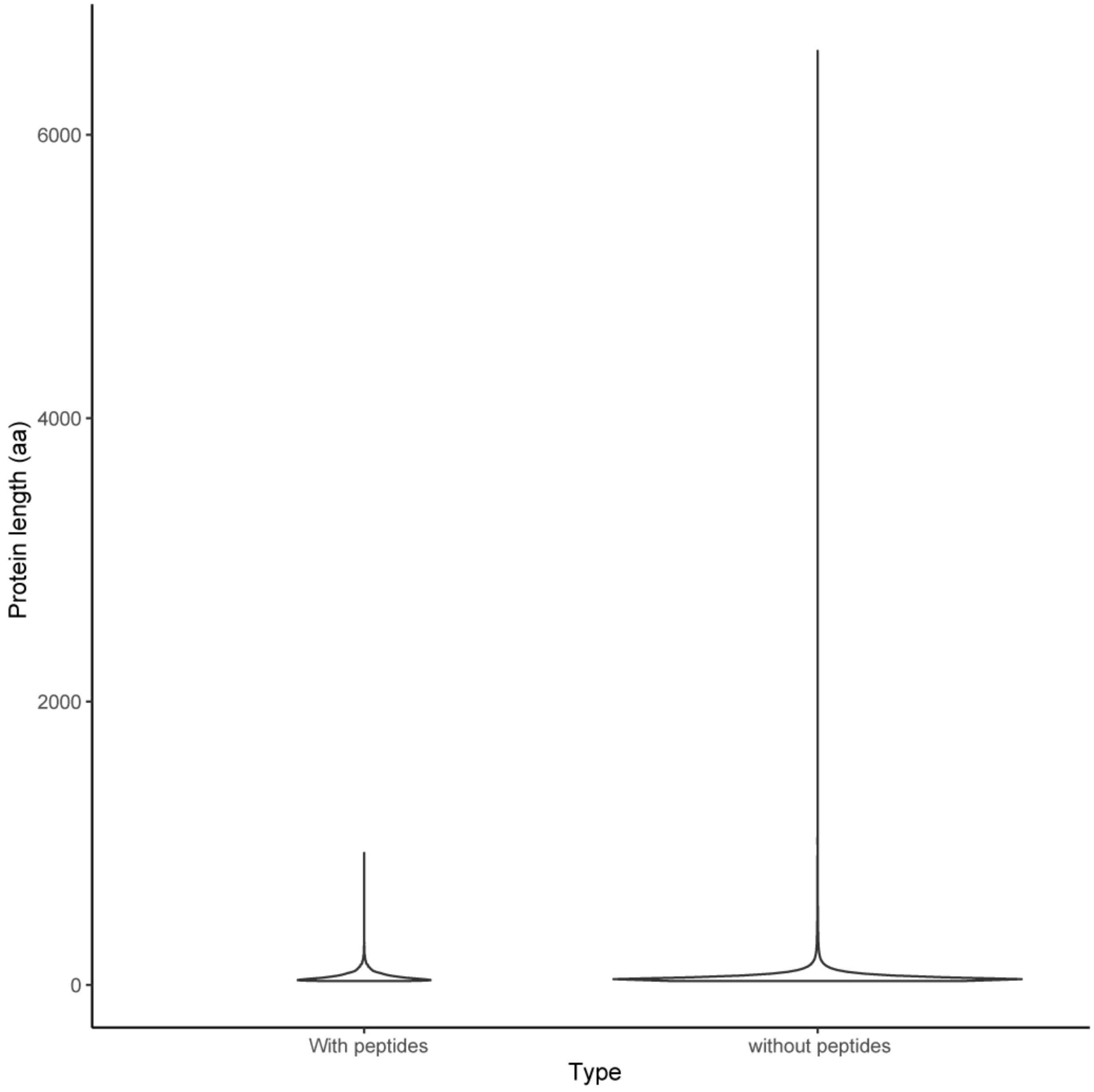

**Supplementary Fig. S4.** Proteins conserved in six or more species are longer in length than proteins that are conserved in five or less species (mean: 92.3 and 63.3 aa respectively, P value 6.58e-25, Wilcoxon rank sum test)

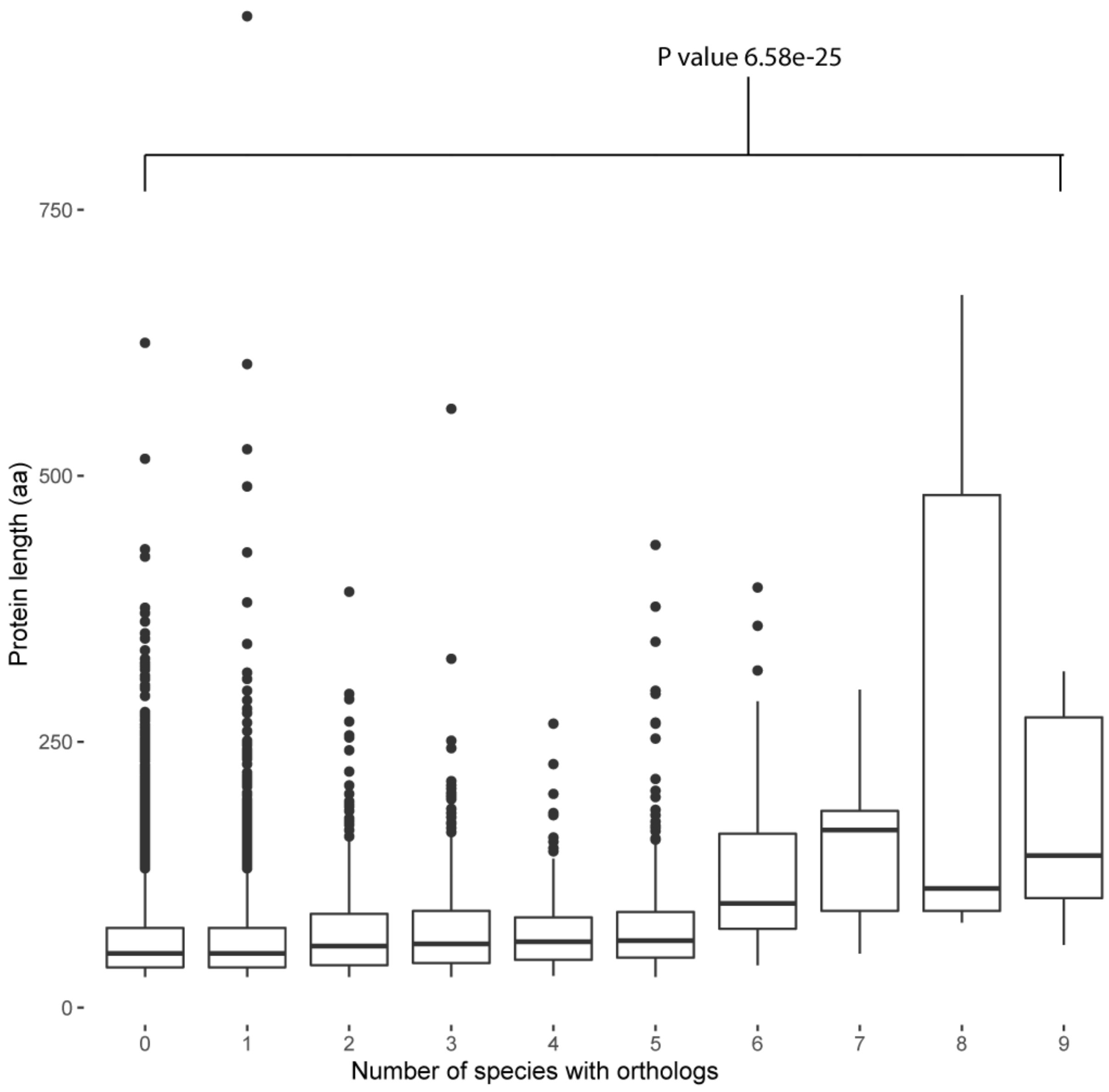

**Supplementary Fig. S5.** An epitope in an out-of-frame ORF in CALCR – a Calcitonin receptor gene- in ovarian cancer (KVFGLNILK), had a high MHC-I binding affinity (8.91 nM) and conserved in five species. Alignment of nucleotide sequences of the ORF across the species is shown.

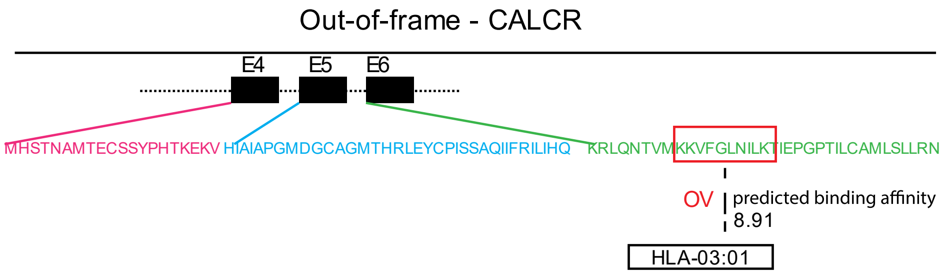


```
Bos           -GGCACAGCACCGATGCTATGACCGAA---TGCAAAAGTTACCCCCATACCAAGGAGAAG     56
Ovis          --GCTCAATATGAATGTT---ACCAGAAAATTATGCAAGACCCTGTTCAACAAACAGAAG     55
Rattus        ATGCTCAGTACAAGTGCTATGACCGGA---TTCAGCAGTTGCCCCCTTATGAGGGAGAAG     57
Pan           ATGCACAGTACAAATGCTATGACCGAA---TGCAGCAGTTACCCGCATACCAAGGAGAAG     57
Mus           ATGCTCAGTACAAATGCTATGACCGGA---TTCATCAGTTGCCCTCTTATGAAGGAGAAG     57
                **:**. *  ..** *   ***..*   * .: .*. : **   : *  *.. *****

Bos           GTCTGTACTGCAACCGTACCTGGGATGGATGGTCCTGCTGGGATGACACACCGGCTGGAG     116
Ovis          GCATTTATTGTAACAGAACCTGGGATGGATGGCTGTGCTGGAATGATGTTGCTGCAGGA-     114
Rattus        GTCCATATTGCAACCGAACTTGGGATGGATGGATGTGCTGGGACGACACTCCAGCTGGTG     117
Pan           GTCCATATTGCAATCGCACCTGGGATGGATGGCTGTGCTGGGATGACACACCGGCTGGAG     117
Mus           GTCTGTACTGCAACCGCACCTGGGATGGCTGGATGTGCTGGGACGACACTCCCGCTGGAG     117
              * .  ** ** ** .* ** ********.***   ******.* ** . : * **:**:

Bos           TG- 118
Ovis          --- 114
Rattus        TA- 119
Pan           TAT 120
Mus           C-- 118
```

**Supplementary Fig. S6**. 73 ncORFs with MHC-bound peptides from genes that have no known or predicted neoantigens from canonical proteins in the studied cancers.

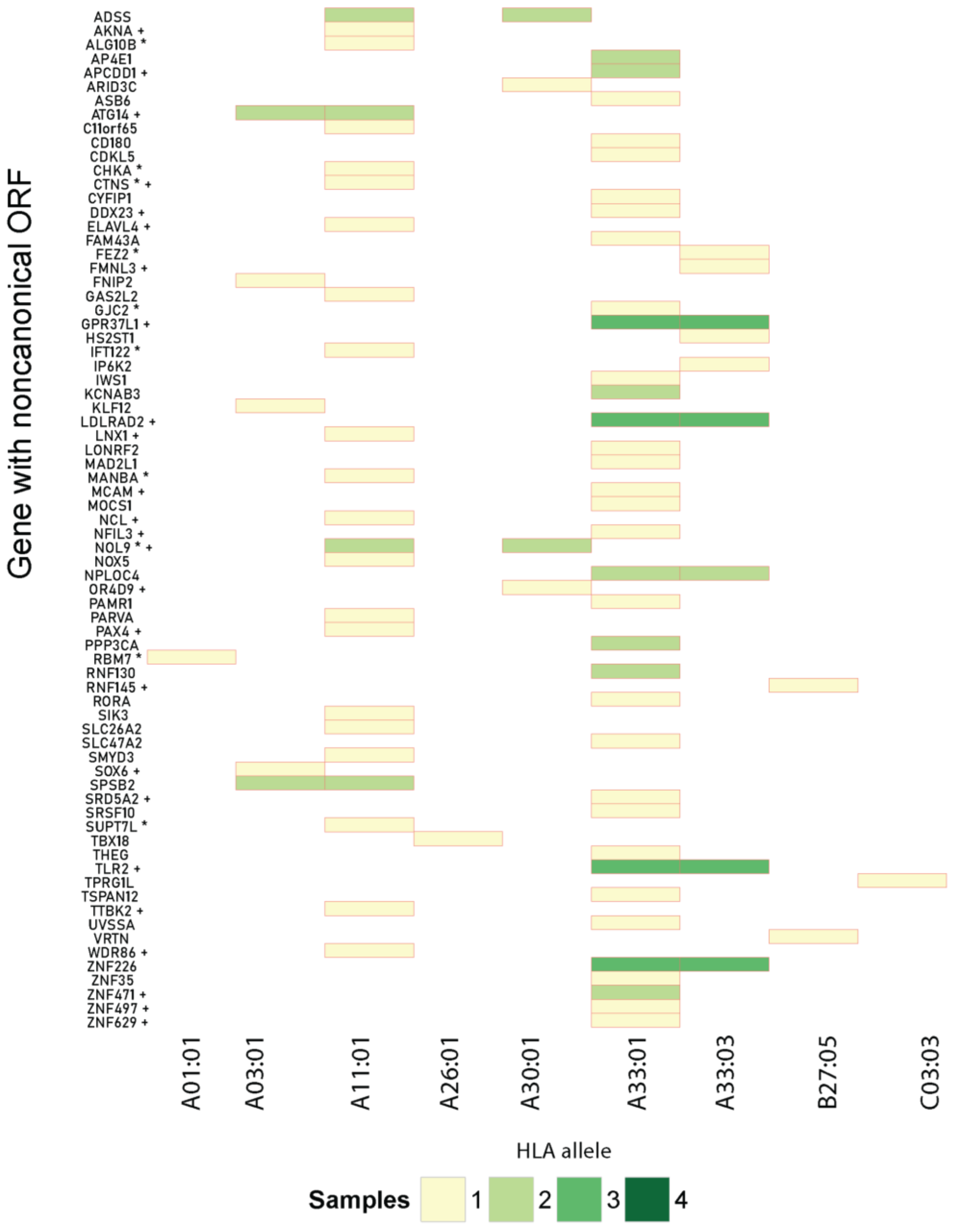

Supplement: zcad024_Supplemental_File [file zcad024_supplemental_file.pdf]
